# Integration of mesopores and crystal defects in metal-organic frameworks via templated electrosynthesis

**DOI:** 10.1038/s41467-019-12268-5

**Published:** 2019-10-02

**Authors:** Xinchen Kang, Kai Lyu, Lili Li, Jiangnan Li, Louis Kimberley, Bin Wang, Lifei Liu, Yongqiang Cheng, Mark D. Frogley, Svemir Rudić, Anibal J. Ramirez-Cuesta, Robert A. W. Dryfe, Buxing Han, Sihai Yang, Martin Schröder

**Affiliations:** 1 School of Chemistry, University of Manchester, Manchester, M13 9PL UK; 2 Beijing National Laboratory for Molecular Sciences, CAS Key Laboratory of Colloid, Interface and Chemical Thermodynamics, Institute of Chemistry, Chinese Academy of Science, Beijing, 100190 China; 3 Oak Ridge National Laboratory, Oak Ridge, TN 37831 USA; 4 Diamond Light Source, Harwell Science Campus, Oxfordshire, OX11 0DE UK; 5 ISIS Facility, STFC Rutherford Appleton Laboratory, Oxfordshire, OX11 0QX UK

**Keywords:** Chemical synthesis, Metal-organic frameworks

## Abstract

Incorporation of mesopores and active sites into metal-organic framework (MOF) materials to uncover new efficient catalysts is a highly desirable but challenging task. We report the first example of a mesoporous MOF obtained by templated electrosynthesis using an ionic liquid as both electrolyte and template. The mesoporous Cu(II)-MOF MFM-100 has been synthesised in 100 seconds at room temperature, and this material incorporates crystal defects with uncoupled Cu(II) centres as evidenced by confocal fluorescence microscopy and electron paramagnetic resonance spectroscopy. MFM-100 prepared in this way shows exceptional catalytic activity for the aerobic oxidation of alcohols to produce aldehydes in near quantitative yield and selectivity under mild conditions, as well as having excellent stability and reusability over repeated cycles. The catalyst-substrate binding interactions have been probed by inelastic neutron scattering. This study offers a simple strategy to create mesopores and active sites simultaneously via electrochemical formation of crystal defects to promote efficient catalysis using MOFs.

## Abstract (web summary)

Incorporating mesopores and active sites into metal-organic framework materials has proven advantageous for their catalytic application, but remains challenging to achieve. Here the authors obtain mesoporous, defect-rich metal-organic frameworks through templated electrosynthesis using ionic liquids as both electrolyte and template.

## Introduction

The application of metal-organic framework (MOF) materials in catalysis is often restricted by the hindered mass transport of substrates through micropores and the lack of accessible active sites^1–5^. Significant efforts have been devoted to the synthesis of mesoporous MOFs to facilitate substrate transport for catalysis^6,7^, and creating crystal defects to serve as active sites^8–10^. The most common approach to create mesopores in MOFs is to construct the framework using elongated ligands and/or large metal clusters via isoreticular chemistry^11,12^. However, the synthesis of extended organic linkers usually involves multi-step reactions, and the resultant MOFs often suffer from limited framework stability on desolvation. Recently, a number of templating methods using supercritical CO_2_, ionic liquids (ILs), and surfactants have been reported for the synthesis of mesoporous MOFs with improved mass transport and catalytic properties^13–15^.

Removal of coordinated solvent molecules (typically water) in MOFs can generate open metal sites as Lewis acids for catalysis^16,17^. However, this strategy has a number of limitations. Many MOF systems are built from metal centres with saturated coordination environments and without coordinated labile solvents. Also, the removal of coordinated solvents from metal centres can have a detrimental effect on the overall stability of the framework in activated MOFs, and generated open metal sites are readily saturated by solvent molecules used in catalytic reactions, thus remaining inaccessible to target substrates. Recently, the introduction of crystal defects into MOFs as active sites has given rise to enhanced gas adsorption and catalytic properties^18^. In general, defects in MOFs can be divided into inherent and intentional defects^19^. The former are formed without targeted design by stacking faults or dislocations as crystals grow^20^, while judicious choice of synthetic conditions, such as choice of temperature, pH, pressure and solvents, can promote the generation of defects during the MOF synthesis^21,22^. Defects can thus be produced intentionally during synthesis or via post-synthetic treatment. For example, acid- or base-treatment, solvent exchange and activation have been shown to create random crystal defects^23,24^. However, few processes offer effective control of the formation of crystal defects in MOFs, and, moreover, the characterisation of crystal defects is difficult to achieve. Integration of mesopores and active sites within crystal defects in MOFs via readily scalable synthetic routes could in principle greatly facilitate their applications in catalysis. In addition, electrosynthesis can be used for materials production at varying scales^25,26^.

Here, we describe the templated electrosynthesis of samples of MFM-100 containing crystal defects. Mesoporous MFM-100 has been successfully obtained by rapid (<100 s) electrosynthesis at room temperature. The IL, 1-octyl-3-methylimidazolium tetrafluoroborate (OmimBF_4_, Supplementary Fig. 1) was used as an electrolyte and it also serves as a template via self-aggregation to create mesopores in the resultant MOF. The structures, morphology and porosity of MFM-100 can be readily controlled by choice of energy input and IL in the synthesis. These MFM-100 materials contain crystal defects [e.g., uncoupled Cu(II) centres as Lewis acid sites], which have been characterised by confocal fluorescence microscopy (CFM), inelastic neutron scattering (INS), electron paramagnetic resonance (EPR) and Fourier-transform infrared (FTIR) spectroscopy. Significantly, the integration of mesopores and active sites within the MFM-100 scaffold has endowed the resultant MOF with exceptional activity for the aerobic oxidation of a range of alcohols to aldehydes in high yield and selectivity. The MFM-100 catalysts also exhibit excellent stability and reusability.

## Results

### Electrosynthesis of MFM-100

**Fig. 1:**
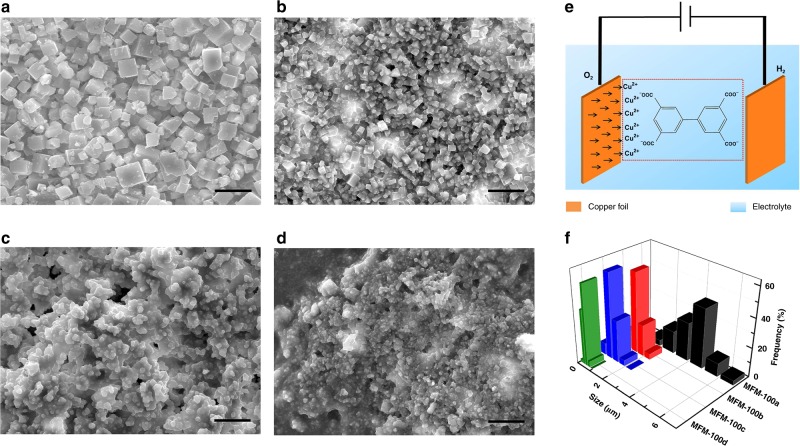
SEM images and particle size distributions of MFM-100 samples. SEM image of MFM-100a (**a**), MFM-100b (**b**), MFM-100c (**c**) and MFM-100d (**d**). **e** Schematic diagram for the electrosynthesis of MFM-100. **f** Particle size distributions for samples of MFM-100. The scale bar in all SEM images is 10 μm. The diameter of IL aggregates is 4 nm

Four samples (denoted as a–d) of MFM-100 (also known as NOTT-100^27,28^) have been synthesised. MFM-100a was synthesised in a solvothermal reaction using Cu(NO_3_)_2_ and biphenyl-3,3′,5,5′-tetracarboxylic acid (H_4_L; Supplementary Fig. 2) in a mixture of DMF/1,4-dioxane/water (2:1:1 v/v/v) at 80 °C for 3 days^27,28^ and isolated as single crystals (yield ~75%) of well-defined morphology (particle sizes ~3–5 microns, Fig. 1a). MFM-100(b,c,d) were obtained by electrosynthesis (Fig. 1b–d) in the same solvent mixture as above using copper foil as both cathode and anode with OmimBF_4_ as the supporting electrolyte (Fig. 1e). During the electrosynthesis, the copper foil at the anode is oxidised to Cu(II) ions, and simultaneously protons from the ligand are reduced to H_2_ under the applied potential, affording deprotonated L^4−^ species that coordinate to Cu(II) ions in the electrolyte to promote the formation of MFM-100. It is worth noting that water reduction is a side-reaction in this process.

We sought to study the effects of temperature and the concentration of IL on the electrosynthesis of MFM-100. MFM-100b was synthesised at 80 °C with 1 wt% of IL in the solvent and isolated as a microcrystalline powder with an average particle size of 1.3 microns (Fig. 1b). MFM-100c and MFM-100d were synthesised using 10 and 50 wt% IL, respectively, in solution at room temperature (25 °C) and were isolated as blue powders of much smaller particle size (average size of 800 and 400 nm for MFM-100c and MFM-100d, respectively) and irregular morphology (Fig. 1c, d). In comparison to MFM-100c, the increased amount of IL in the synthesis of MFM-100d resulted in reduced particle size, presumably because IL can act as a protecting agent and mediate nucleation during crystal growth. The size distributions of these different MOF particles have been obtained by SEM/TEM measurements (Fig. 1f and Supplementary Fig. 3). It is worth noting that no precipitate was formed in the synthesis of MFM-100 at 25 °C in the absence of an applied voltage over 72 h. Interestingly, the electrosynthesis of MFM-100(b, c, d) can be achieved within 100 s in a yield of ~30–40%. A yield of >90% can be achieved at 500 s at room temperature for MFM-100c and MFM-100d, offering a highly efficient and readily scalable route for MOF production.

### Structural analysis and characterisation of MFM-100

**Fig. 2:**
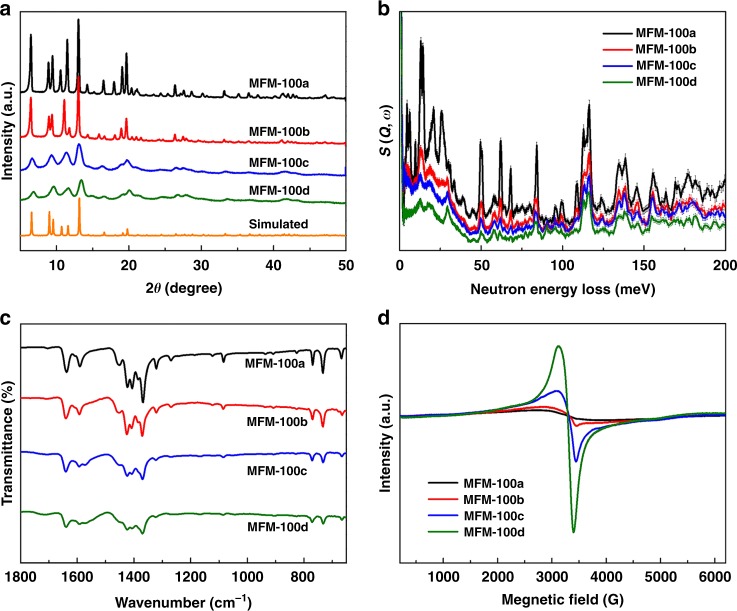
Characterisation of samples of MFM-100. Comparisons of **a** PXRD patterns; **b** INS spectra; **c** FTIR spectra; **d** solid-state EPR spectra at same sensitivity and concentration of MOF material

The crystal structure of MFM-100 is shown in Supplementary Fig. 4. Analysis of the samples by powder X-ray diffraction (PXRD) confirms that all Bragg peaks observed correspond to MFM-100 (Fig. 2a)^27^. MFM-100(a,b) are more crystalline than MFM-100(c,d) suggesting that the latter have substantial crystal defects, leading to reduction in long-range order of the solid-state lattice. Vibrational spectroscopy is a commonly used technique to characterise crystal defects^19^. Both INS and FTIR spectroscopy confirm full retention of vibrational features in the electrosynthesised samples of MFM-100 (Fig. 2b, c). The INS features of MFM-100c and MFM-100d are heavily convoluted and broadened, and FTIR spectra show notable decrease in intensity and increase of bandwidth on going from MFM-100a to MFM-100d. These results are consistent with the reduced crystallinity and presence of crystal defects in MFM-100(c,d). A small increase in intensity at 1574 cm^−1^ (assigned to the C=C vibration of the imidazole ring; Supplementary Fig. 5) is observed in the FTIR spectra of MFM-100c and MFM-100d, indicating the presence of trace IL cations, which has been confirmed also by elemental analytical data (Supplementary Table 1). Neither B nor F was detected by ICP-OES or X-ray photoelectron spectroscopy (XPS) analysis, suggesting the absence of IL anions in the framework. Measurements of the contact angle at the MOF to water interface confirm 32.5°, 35.9°, 46.4° and 55.4° for samples MFM-100(a,b,c,d), respectively, consistent with the presence of the Omim^+^ cations in the electrosynthesised samples leading to increased hydrophobicity, which is a highly desirable feature to improve the stability of water-sensitive MOFs (Supplementary Fig. 6). Full chemical analysis of these materials has revealed that the stoichiometry of MFM-100 (i.e., the metal-to-ligand ratio) decreases on going from MFM-100a (1.99:1) to MFM-100d (1.79:1), consistent with the partial replacement of Cu(II) by Omim^+^ (Supplementary Table 1). All four MOFs were digested in hydrochloric acid and the molar ratio of Omim^+^/L^4−^ in solution analysed by ^1^H nuclear magnetic resonance (NMR) spectroscopy (Supplementary Table 1). This affords highly consistent results with the chemical analytical data.

The EPR spectrum of binuclear paddlewheel Cu(II)–Cu(II) species shows only a low intensity signal due to anti-ferromagnetic coupling between the metal centres. This is reflected in the EPR spectra of the solid-state samples of MFM-100a to MFM-100d (Fig. 2d) in which a progressively enhanced signal centred at 3250G is observed confirming the presence of notable amounts of uncoupled Cu(II) centres as defects in the framework of MFM-100c and MFM-100d, with an EPR peak width of ~300G^29^. Thermogravimetric analysis (TGA) suggests that MFM-100c and MFM-100d have lower decomposition temperatures (300 °C) than MFM-100a (340 °C) and MFM-100b (320 °C) (Supplementary Fig. 7), consistent with the presence of incomplete metal–ligand coordination in the defective MOFs. The TGA plots of MFM-100c and MFM-100d further confirm the absence of encapsulated free IL molecules within the MOF pores, consistent with the chemical analysis.

**Fig. 3:**
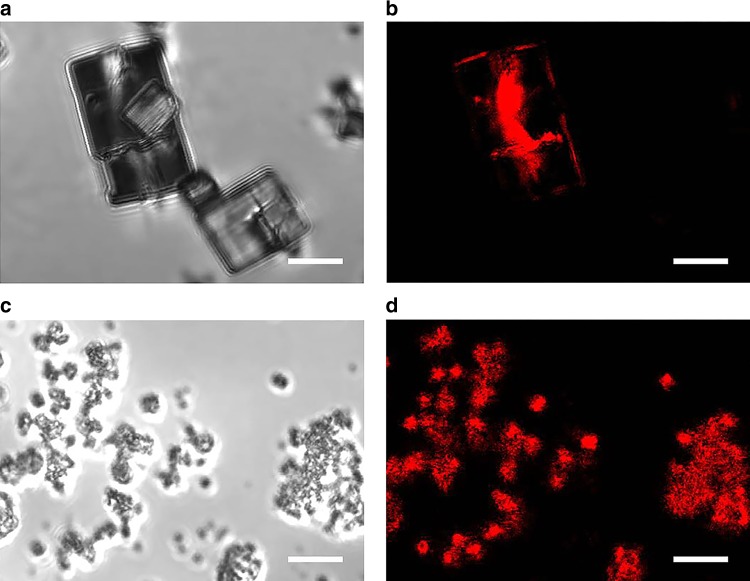
Micrographs and CFM images. Views of MFM-100a **a** micrograph, **b** CFM and of MFM-100d **c** micrograph, **d** CFM. The scale bar is 5 μm in all images. The fluorescence (shown in red colour) indicates the presence of crystal defects determined by the oligomerisation of furfuryl alcohol

Interestingly, it has been recently reported that the introduction of defects as Lewis acid sites in the MOF lattice can be captured by CFM using an acid-catalysed furfuryl alcohol probe reaction^30^. The microphotographs and CFM images reveal a stark comparison between MFM-100a and MFM-100d (Fig. 3). The CFM images can locate emissive products from the oligomerisation of furfuryl alcohol catalysed by Lewis acid sites. MFM-100a only shows fluorescence response at the crystal boundaries (i.e., at edges and gaps), and the absence of mesopores and internal structural defects in MFM-100a is further confirmed by 3D scan images of a single crystal of MFM-100a at different Z depths (Supplementary Fig. 9). In contrast, all particles of MFM-100d exhibit strong fluorescence that is distributed evenly across the entire particle, directly confirming the presence of homogenous active Cu(II) sites as Lewis acid defects within MFM-100d.

### Analysis of porosity in MFM-100

The porosity of the MFM-100 samples was studied by N_2_ isotherms at 77 K (Table 1 and Supplementary Figs. 10 and 11) and the presence of mesopores analysed by the Barrett–Joyner–Halenda (BJH) model^7^. As expected, the highly crystalline MFM-100(a,b) samples show high surface areas (1507–1586 m^2^ g^−1^) that are consistent with the previously reported values^27^; the pore size distribution is centred at 6.5 Å and there is an absence of mesopores. MFM-100c shows a total Brunauer–Emmett–Teller (BET) surface area of 1486 m^2^ g^−1^ and a small amount of mesopores (*V*_meso_ *=* 0.41 cm^3^ g^−1^ and S_meso_/S_total_ = 0.27). In contrast, mesopores centred at 4.7 nm are observed in MFM-100d (*V*_meso_ *=* 1.33 cm^3^ g^−1^ and *S*_meso_/*S*_total_ = 0.69), which has a total BET surface area of 1544 m^2^ g^−1^. Aggregates of Omim^+^-based ILs have an average size of ~4 nm^31^, which matches precisely the dimensions of mesopores in MFM-100(c,d), confirming the templating effect of IL in the electrosynthesis. The size of IL aggregates relies on the length of alkyl chain and longer alkyl chains result generally in larger aggregates^31^. OmimBF_4_ has thus been selected to match the mesopores in the resultant MOFs. The total surface area is observed to decrease slightly with increase of crystal defects because of the collapse of micropores.

**Table 1:** Summary of the porosity of all MOFs in this study

Entry	Samples	*S*_total_ (m^2^ g^−1^)	*S*_meso_ (m^2^ g^−1^)	*D*_meso_ (nm)	*V*_meso_ (cm^3^ g^−1^)
1	MFM-100a	1507	142	–	–
2	MFM-100b	1586	170	–	–
3	MFM-100c	1486	399	4.6	0.41
4	MFM-100d	1544	1069	4.7	1.33
5	HKUST-1a	1017	46.6	–	–
6	HKUST-1b	719	581	4.9	0.71
7	MOF-2a	196	214	4.3	0.23
8	MOF-2b	214	236	5.1	0.31

*S*_total_ = total surface area obtained from the BET model; *S*_meso_ = BJH desorption cumulative surface area of pores; *D*_meso_ = mesopore size obtained from the BJH model; *V*_meso_ = mesopore volume obtained from the BJH model. “–” indicates negligible values

### Synthesis of MFM-100 under different conditions

To confirm further the critical role of electrosynthesis and IL in the synthesis of defective and mesoporous MOFs, MFM-100(b’–d’) were synthesised using the same conditions as MFM-100(b–d), but in the absence of an applied potential. The solvothermal synthesis was carried out over 3 days to give the samples MFM-100(b’–d’). These materials show improved crystallinity by PXRD and similar morphology and particle size distributions compared with MFM-100(b–d) (Supplementary Figs. 12 and 13), indicating that defects were generated primarily by the rapid electrosynthesis. In addition, MFM-100d” was prepared by electrosynthesis at 8 V for 3 h in the absence of an IL at room temperature. MFM-100d” shows ordered cubic morphology with highly crystalline structure and there is an absence of mesopores (Supplementary Figs. 14–16), indicating that the IL plays a crucial role in creating defects and mesopores. The effect of potential was also investigated via the synthesis of MFM-100d_6V_ and MFM-100_10V_ at an applied potential of 6 and 10 V, respectively. Overall, the crystallinity of the resultant MOF increases and the particle size reduces with increasing potential (Supplementary Figs. 17 and 18).

### Synthesis and characterisation of selected Cu(II)-based MOFs

The Cu(II)-based MOFs, HKUST-1 and MOF-2^32^, have also been prepared by solvothermal synthesis (denoted as HKUST-1a and MOF-2a) and by templated electrosynthesis (denoted as HKUST-1b and MOF-2b) as described above (see Methods for details). The micropore sizes of HKUST-1 and MOF-2 are 6.8 and 13.1 Å, respectively. Analysis of the structure and porosity of these samples confirms that the electrosynthesised MOFs show reduced crystallinity and the presence of significant amounts of mesopores (Table 1 and Supplementary Figs. 19 and 20) compared with the samples obtained by solvothermal synthesis. This suggests that the strategy based upon templated electrosynthesis developed here has general applicability to the synthesis of mesoporous, defective Cu(II)-based MOFs.

### Catalytic activity of Cu(II)-based MOFs for alcohol oxidation

Aldehydes are key intermediates and high-value chemicals in the perfume and pharmaceutical industries^33,34^. Oxidation of alcohols over Cu(II)-based catalysts is an efficient method to synthesise aldehydes^7,35–37^, and we therefore tested the catalytic activity of MFM-100, HKUST-1 and MOF-2 for the oxidation of a range of primary and secondary alcohols using 2,2,6,6-tetramethyl-piperidine-1-oxyl (TEMPO) as a co-catalyst (Table 2). There was no conversion of benzyl alcohol in the absence of MOF catalyst or using IL as the catalyst. Oxidation of benzyl alcohol to benzaldehyde was undertaken using solvothermally prepared catalyst. MFM-100a gave a yield of 40% and exhibited higher activity than HKUST-1a (yield = 19%) and MOF-2a (yield = 34%), indicating that MFM-100 is a better candidate for alcohol oxidation. Interestingly, the yield of benzaldehyde increases on going from MFM-100a (40%) to MFM-100d (>99%). Similarly, HKUST-1b and MOF-2b show better catalytic performance than HKUST-1a and MOF-2a. Importantly, MFM-100d shows leading catalytic performance for the oxidation of alcohols compared with a range of reported Cu(II)-MOFs (Supplementary Table 3). The catalytic performance of other samples of MFM-100 is summarised in Supplementary Table 2. It is widely accepted that particle size has a strong influence on the catalytic performance. Compared with MFM-100d, MFM-100d’ shows notably reduced catalytic activity (yield = 61%). In addition, MFM-100d_10V_, showing smaller particle size than MFM-100d, also gave a reduced yield of benzylaldehyde (90%). Thus, crystal defects consisting of uncoupled Cu(II) centres in MFM-100(c,d) play a critical role in the observed catalytic activity, leading to the quantitative conversion of benzyl alcohol with MFM-100d.

**Table 2:** Summary of the oxidation of selected alcohols to aldehydes using Cu(II)-MOFs as catalysts^*^

^*^Reaction conditions: 0.1 mmol catalyst (based on Cu content from ICP); 1 mmol alcohol, 0.1 equiv TEMPO; 0.2 equiv K_2_CO_3_; 4 mL solvent (entries 1–12 MeCN, 13–24 DMF); 1 atm O_2_; 75 °C

^†^Kinetic diameter of width and length of different compounds were measured by GaussView software

^┴^There is no conversion in the absence of MOF catalyst for all substrates

^‡^Average yield of aldehyde. All experiments were performed three times

To investigate the effect of pore size (and hence mass transport) on catalytic activity, the oxidation of a larger substrate, salicin, was tested over all catalysts. Salicin is widely presented in bark and leaves of various species of plants, and the transformation of salicin into useful chemicals is a promising target in biomass conversion^38^. MFM-100d with an increased amount of mesopores (*V*_meso_ = 0.39 and 1.17 cm^3^ g^−1^ for MFM-100c and MFM-100d, respectively) exhibits significantly improved yields of aldehyde (46% and >99% for MFM-100c and MFM-100d, respectively), demonstrating the critical role of mesopores on the transport and hence conversion of bulky substrates. In comparison, HKUST-1a and MOF-2a both show poor activity for this conversion. Interestingly, mesoporous HKUST-1b, which has similar pore size as MFM-100d, shows relatively poor catalytic activity due to fewer defects and more crystalline framework structure (Supplementary Fig. 19a). The strong fluorescence in CFM images originates from the oligomerisation product of furfuryl alcohol, the size of which is similar to that of benzyl alcohol. The homogenous distribution of the fluorescence across the entire MOF crystal indicates that the reaction occurs both within the MOF pores and on the surface of particles.

**Fig. 4:**
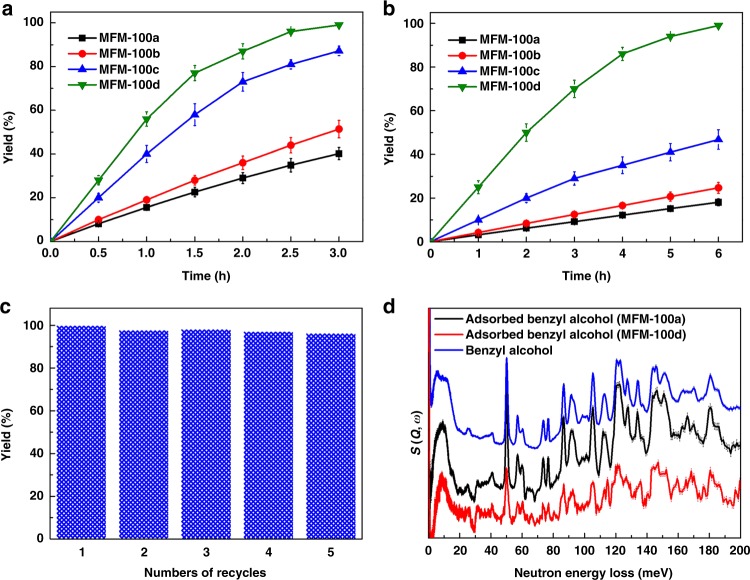
Time-dependent yields of aldehydes from selected substrates catalysed over MFM-100. Yields of oxidation of **a** benzyl alcohol (4.3 × 7.5 Å^2^) and **b** salicin (7.0 × 9.5 Å^2^). **c** Reusability of MFM-100d (entry 4 in Table 2). **d** Comparison of INS spectra of solid-state and adsorbed benzyl alcohol in MFM-100a and MFM-100d. The error bars in **a** and **b** were obtained by repeating each reaction three times

The oxidation of thiophen-2-ylmethanol and 3,3′,5,5′-tetrakis(trifluoromethyl)benzhydrol of distinct molecular sizes (3.6 × 6.8 and 7.0 × 10.0 Å^2^, respectively) was also studied (Table 2). As before, MFM-100(c,d) exhibit notably higher activity than MFM-100(a,b) and MFM-100d affords quantitative production of the corresponding aldehydes in both cases. The time-dependence of the formation of aldehyde further confirmed the critical effect of pore size on substrate selectivity (Fig. 4a, b). Significantly, although with lower dosage of TEMPO and base, MFM-100d shows higher activity for alcohol oxidation than all previously reported Cu(II)-MOFs^7,39,40^. These results confirm that integration of mesopores and uncoupled Cu(II) sites in MFM-100d results in its exceptional catalytic performance.

The reusability of MFM-100d was studied for the oxidation of benzyl alcohol. The catalyst shows excellent stability over five repeated cycles of reaction without detectable loss of activity (Fig. 4c). To test the possible leaching of any catalytic active sites into solution, the catalyst was removed via hot filtration at an alcohol conversion of 50%, and no notable further reaction was found to proceed using the solvate, confirming the absence of leaching of catalytically-active Cu(II) ions during the reaction. The bound Omim^+^ cations in MFM-100d increases the framework hydrophobicity and leads to high stability in polar solvents such as MeCN and DMF. Used MFM-100d was separated from solution and characterised by SEM, PXRD, FTIR and N_2_ adsorption/desorption isotherms at 77 K (Supplementary Fig. 21). Little change was observed compared with that of a fresh sample, demonstrating the excellent stability of MFM-100d for the oxidation of alcohols.

### Vibrational analysis of host-guest binding dynamics

INS has been applied to investigate the host–guest binding interaction between MFM-100a and MFM-100d and benzyl alcohol. The excellent agreement between the density functional theory (DFT)-calculated and experimental INS spectra for solid benzyl alcohol allowed full assignment of its vibrational modes (Supplementary Fig. 22). Significant increases in the peak intensity of INS spectra of MFM-100(a,d) were observed upon adsorption of benzyl alcohol. Comparison of the difference INS spectrum (i.e., signals for the adsorbed substrate together with changes in the local MOF environment) with that of solid benzyl alcohol revealed a number of changes (Fig. 4d). The peaks at low energy (<20 meV), assigned to the translational and rotational modes of benzyl alcohol, shift to lower energy with a continuum profile, indicating the adsorbed benzyl alcohol molecules are disordered on the pore surface and have hindered motion owing to the binding to the surface sites. The INS peak at 162 meV (assigned to the wagging mode of the –OH group in benzyl alcohol) almost disappears on adsorption in MFM-100d, indicating deprotonation of the alcohol on the MOF surface. The peaks at 135 and 152 meV (assigned to the wagging and twisting modes of the –CH_2_– group, respectively) shift to higher energies consistent with the formation of benzyl alcohol anion on the MOF surface, which leads to its activation and subsequent oxidation.

### Summary

We report the use of templated electrosynthesis to integrate mesopores with crystal defects in Cu(II)-based MOFs. This enables the production of MFM-100 within 100 s at room temperature, and the structures, morphology, porosity and crystal defects of the resultant materials can be readily controlled by the use of ILs as templates in the synthesis. The presence and nature of crystal defects in MFM-100 have been characterised comprehensively by CFM, INS, EPR, FTIR and chemical analysis, and these materials show excellent catalytic activity and stability for the oxidation of a range of alcohols, including inactive primary alcohols. We found that the co-presence of crystal defects and mesopores greatly promotes catalytic oxidation with exceptional catalytic performance observed for MFM-100d. The method developed here paves a new and easy-scalable pathway to synthesise mesoporous, defect MOFs as efficient catalysts for alcohol oxidation.

## Methods

### Solvothermal synthesis of MOFs

MFM-100a was synthesised following the reported method^27,28^. In a typical experiment, Cu(NO_3_)_2_·2.5H_2_O (0.20 g, 0.86 mmol) and biphenyl-3,3′,5,5′-tetracarboxylic acid (H_4_L) (0.10 g, 0.30 mmol) were mixed in DMF/dioxane/H_2_O (2:1:1 v/v/v) (75 mL) containing hydrochloric acid solution (37%) (0.5 mL). The solution was placed in an autoclave and heated at 80 °C for 3 days. The as-synthesised material was collected via filtration and washed with hot DMF and acetone, each for five times, and dried at 60 °C. HKUST-1a and MOF-2a were synthesised similarly in autoclaves at 60 and 110 °C, respectively^32^.

### Electrosynthesis of MOFs

All syntheses were performed on a CHI660E electrochemical workstation. In a typical experiment, H_4_L (0.05 g, 0.15 mmol) and a fixed amount (1, 10 and 50 wt% for MFM-100b, MFM-100c, and MFM-100d, respectively) of IL were mixed in a stirred solution of DMF/dioxane/H_2_O (2:1:1 v/v/v) (75 mL) containing hydrochloric acid solution (37%) (0.5 mL) at 80 °C for MFM-100b and at 25 °C for MFM-100c and MFM-100d for 10 min. The resultant clear solution was used as the electrolyte. Copper foils (2 × 1 cm^2^) were used as both cathode and anode, which were immersed into the electrolyte, and the electrolysis conducted under an applied potential of 8 V (Fig. 1e). A blue precipitate formed rapidly as the electrolysis proceeded for 100 s. The electrolysis was continued for a further 400 s, and the precipitate collected via filtration and washed with DMF and acetone, each for five times, and the material dried at 60 °C. Electrosynthesis of HKUST-1b and MOF-2b were carried out using the same condition as MFM-100d except using trimesic and terephthalic acid as linkers.

### Material characterisations

PXRD data were collected on the X-ray diffractometer (Model D/MAX2500, Rigaka) with Cu-Kα radiation at a scan speed of 2 °C/min. FTIR data were collected on a Bruker Vertex spectrometer on Beamline B22 at Diamond Light Source, while TGA measurements were performed under N_2_ at a heating rate of 10 °C/min. Contact angle measurements between MOF and a water droplet were obtained using Biolin Attension goniometer, and the porosity of these materials was studied by adsorption-desorption isotherms for N_2_ using a Micromeritics ASAP 2020M system. Prior to the BET measurement, all samples were activated under dynamic vacuum at 130 °C for 12 h. The BJH method and the Horvath–Kawazoe method were used for analysis of mesopores and micropores, respectively. The morphologies of the materials were characterised by SEM on a Quanta 650 and TEM on a FEI Tecnai T20.

### Samples preparation and measurement of CFM

The procedure of sample preparation and measurement of CFM data was same as that reported previously^30^. A 10 mg sample of MOF was immersed in furfuryl alcohol (1 mL) at 80 °C for 24 h. After this incubation step, the MOF was collected by filtration and dried. An Olympus Fluoview FV-1000 instrument was used to record fluorescence micrographs.

### EPR measurements

The EPR spectra arising from solid MFM-100 samples were recorded in continuous-wave at 9 GHz (X-band) using a Bruker Micro spectrometer at room temperature with a microwave power of 2 mW, and the spectra reported herein were typically the average of 20 scans. The intensity of the EPR signal of different samples was normalised to the sample quantity.

### INS experiments and DFT calculations

INS spectra were recorded on the TOSCA spectrometer at the ISIS Facility at the STFC Rutherford Appleton Laboratory^41^. TOSCA is an indirect geometry crystal analyser instrument that provides a wide dynamic range (16–4000 cm^−1^) with resolution optimised in the 50–2000 cm^−1^ range. In this region TOSCA has a resolution of 1.25% of the energy transfer. For background tests, samples were loaded into an in situ catalysis cell with a copper vacuum seal and connected to a gas handling system. The sample was heated at 100 °C for 12 h under He flow to remove any remaining trace water before the experiment. The samples were cooled to below 15 K by a closed cycle refrigerator cryostat for data collection^42^. The MOF sample (0.7 g) was dosed with benzyl alcohol (0.5 g) and sealed in the cell, which was then heated at 100 °C for 24 h to reach equilibrium. Data collection was conducted in the same way as the background collection. DFT calculations were performed using CASTEP^43^. The generalised gradient approximation, as implemented by Perdew–Burke–Ernzerhof, was used to describe the exchange-correlation interactions. Ultra-soft pseudopotentials were employed to account for the effects of core electrons. Tkatchenko–Scheffler dispersion correction was used for van der Waals interactions^44^. Energy cutoff for plane-wave basis was 450 eV, and the unit cell configuration determined by XRD was used as the initial structure for the simulations. The atomic coordinates were relaxed to allow minimisation of the potential energy and the interatomic forces. The energy tolerance for the electronic structure calculations was 5 × 10^−10^ eV, for ionic relaxation 5 × 10^−9^ eV and the tolerance for the interatomic forces was 1 meV/Å. After convergence was reached, the dynamical matrix was obtained using the finite displacement method, from which the phonon frequencies and vibrational modes were calculated. The electronic structure calculations were performed on the Γ-point only for the MOF due to its large unit cell (crystal structure: this work), and on a 5 × 7 × 3 Monkhorst–Pack mesh for benzyl alcohol (crystal structure: CCDC 725239). The supercell used for the phonon calculation was 1 × 1 × 1 of the unit cell for the MOF, and 2 × 3 × 1 of the unit cell for benzyl alcohol. Phonon sampling in the Brillouin zone was performed on a 3 × 3 × 2 Γ-centred mesh for the MOF, and 7 × 9 × 5 Monkhorst–Pack mesh for benzyl alcohol. The OClimax software was used to convert the DFT-calculated phonon results to the simulated INS spectra^45^.

### Oxidation of alcohols

All oxidation reactions were carried out in three-necked flasks using similar protocols. The oxidation of benzyl alcohol is described here in detail. Benzyl alcohol (108 mg, 1 mmol), MOF (0.1 mmol, based on the Cu content from ICP), 0.1 equiv TEMPO and 0.2 equiv K_2_CO_3_ (pellets of diameter of ~1 mm) were loaded into 4 mL of MeCN. The air in the flask was replaced by O_2_, and the flask immersed in an oil bath at 75 °C. The O_2_ at ambient pressure was supplied by a balloon of O_2_. After reaction, the mixture was centrifuged and the solution taken at the targeted reaction time and analysed by GC and ^1^H NMR spectroscopy. Salicin and the corresponding aldehyde were analysed by ^1^H NMR spectroscopy and other reactants and products analysed by gas chromatography (Supplementary Fig. 23).

### Stability of the catalyst

The reaction mixture was centrifuged and the solid MOF catalyst containing K_2_CO_3_ was collected, washed with acetone, dried at 60 °C for 24 h, and re-used for the next run. Five cycles of repeated tests were conducted. For the leaching test, the reaction mixture at 50% conversion and the supernatant were checked for reactivity with fresh K_2_CO_3_. After the catalysis, K_2_CO_3_ and MFM-100d catalyst were separated from the suspension for further characterisation.

## Supplementary information

Supplementary Information

## Data Availability

The data that support the findings of this study are available from the corresponding authors on reasonable request.
