# Supplementary material for: Integration of mesopores and crystal defects in metal-organic frameworks via templated electrosynthesis
Source: Nat Commun. 2019 Oct 2;10:4466. doi: 10.1038/s41467-019-12268-5 (PMC6775123; doi:10.1038/s41467-019-12268-5)
Supplement: Supplementary file 1 — Supplementary Information [file 41467_2019_12268_MOESM1_ESM.pdf]

Supplementary Information

Integration of mesopores and crystal defects in metal-organic frameworks *via* templated electro-synthesis

Kang et al.

Supplementary Methods

Materials. $OmimBF_4$ (>99%) was purchased from Lanzhou Yulu Fine Chemical Co., Ltd. Copper foil (>99.99%), $Cu(NO_3)_2·2.5H_2O$ (>99%), biphenyl-3,3',5,5'-tetracarboxylic acid (H_4L, >99%), hydrochloric acid (37%), acetonitrile (>99%), N,N-Dimethylformamide (DMF, >99%), 1,4-dioxane (>99%), hydrochloride acid (37%), trimesic acid (H_3BTC, >99%), terephthalic acid (H_2BDC, >99%), 2,2,6,6-tetramethylpiperidine 1-oxyl (>98%), K_2CO_3 pearls (>99%), furfuryl alcohol (>99%), benzyl alcohol (>99%), salicin (>99%), thiophen-2-ylmethanol (>98%) and 3,3',5,5'-tetrakis(trifluoromethyl)benzhydrol (>97%) were purchased from Sigma-Aldrich Co., UK.

Supplementary Figures

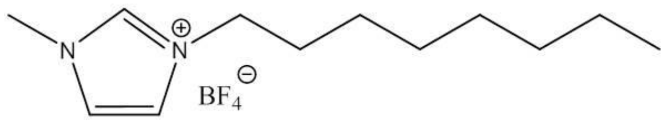

Supplementary Fig. 1. The structure of $OmimBF_4$.

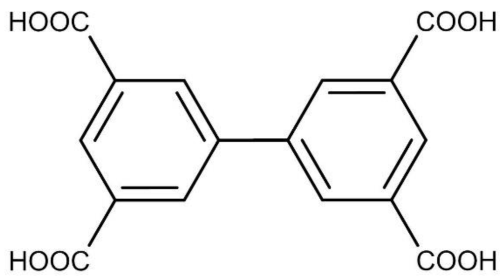

Supplementary Fig. 2. The structure of the ligand in MFM-100.

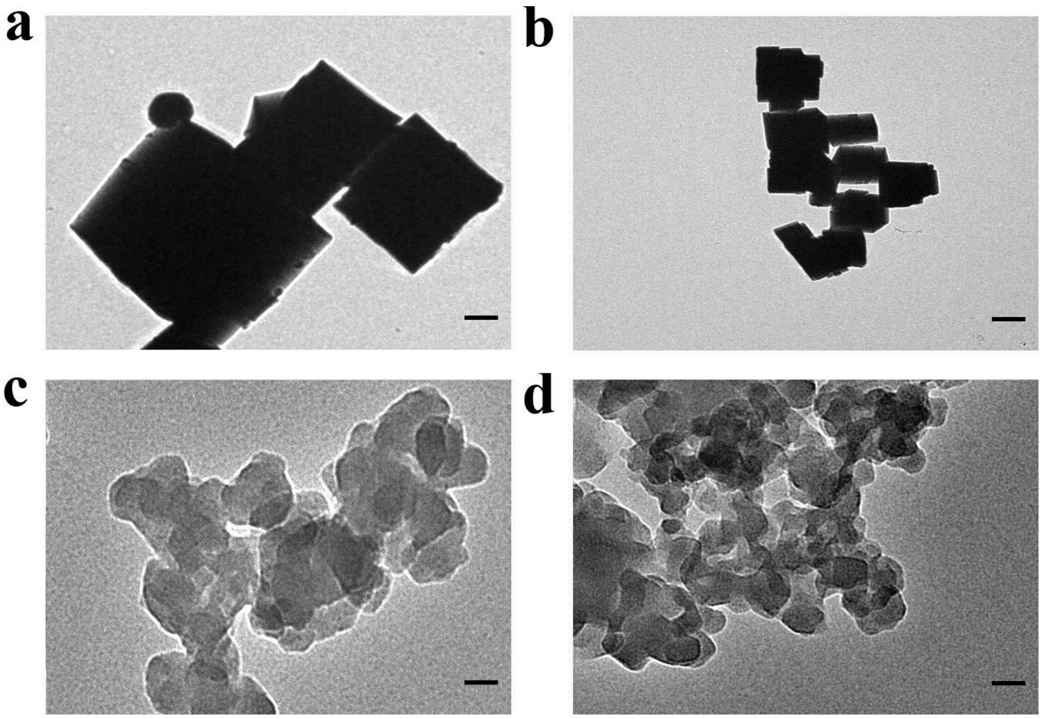

Supplementary Fig. 3. TEM images of MFM-100. (a) MFM-100a; (b) MFM-100b; (c) MFM-100c; (d) MFM-100d. The scale bars are 1 μm in (a, b) and 500 nm in (c,d).

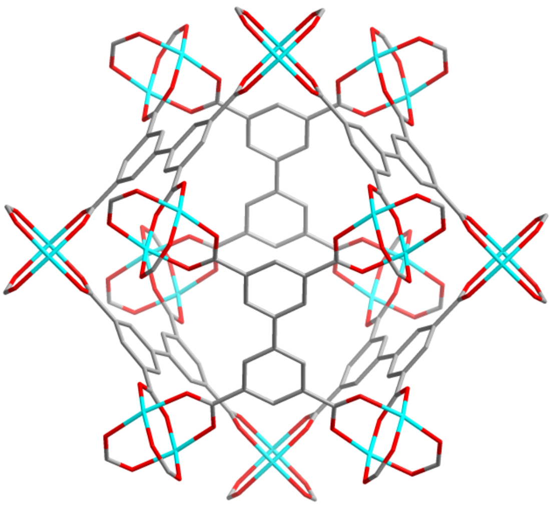

Supplementary Fig. 4. View of crystal structure of MFM-100 (Cu: blue, C: grey, O: red). Hydrogen atoms are omitted for clarity.

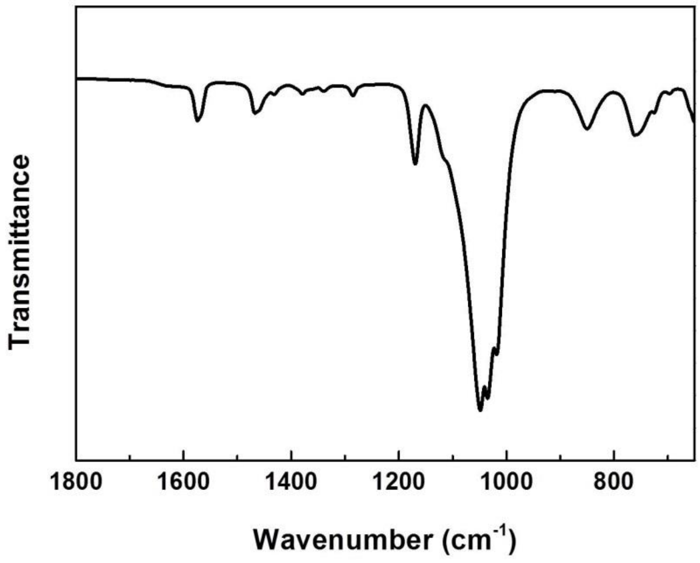

Supplementary Fig. 5. FTIR spectrum of $OmimBF_4$.

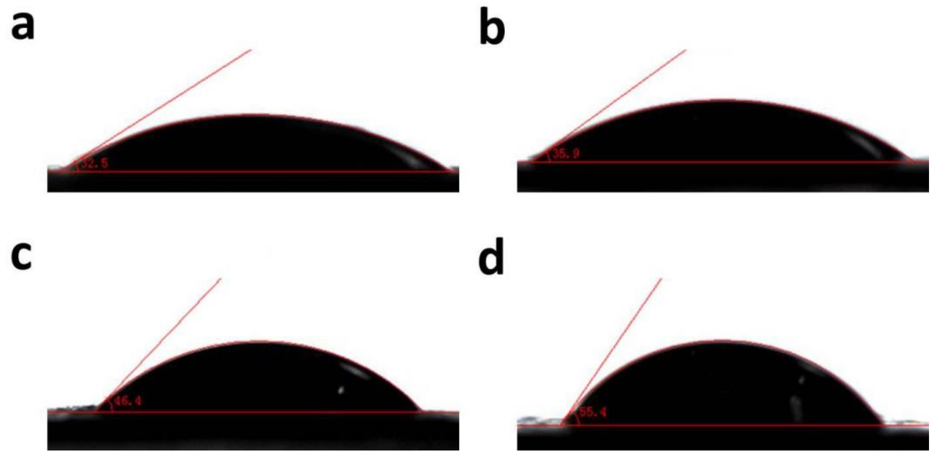

Supplementary Fig. 6. Contact angles between surfaces of MFM-100 samples and water droplet in air. (a) MFM-100a; (b) MFM-100b; (c) MFM-100c; (d) MFM-100d.

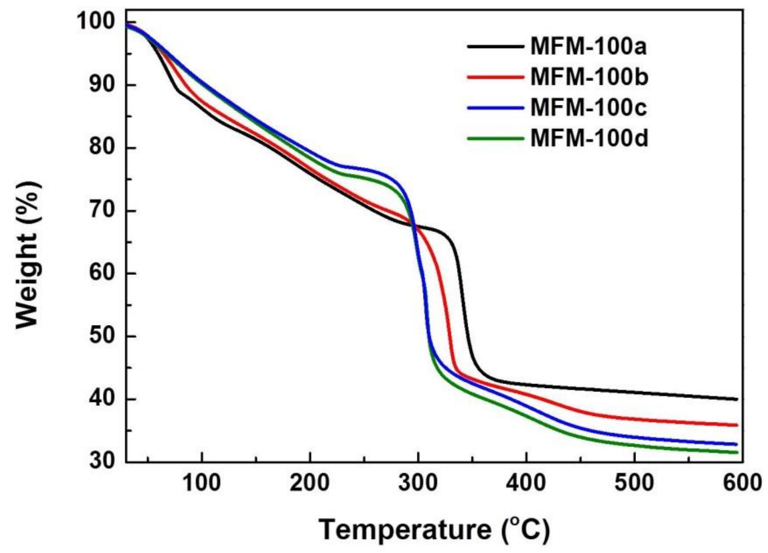

Supplementary Fig. 7. TGA curves of MFM-100 under N_2 atmosphere.

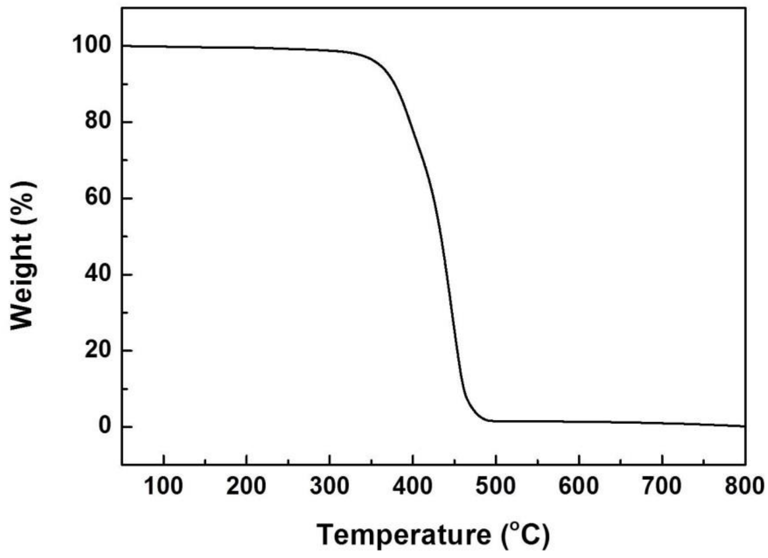

Supplementary Fig. 8. TGA curve for $OmimBF_4$. No obvious weight loss was observed in the TGA curve for MFM-100d, which contains the $Omim^+$ in the framework. This suggests that the $Omim^+$ cation is bound with the framework consistent with the chemical analysis.

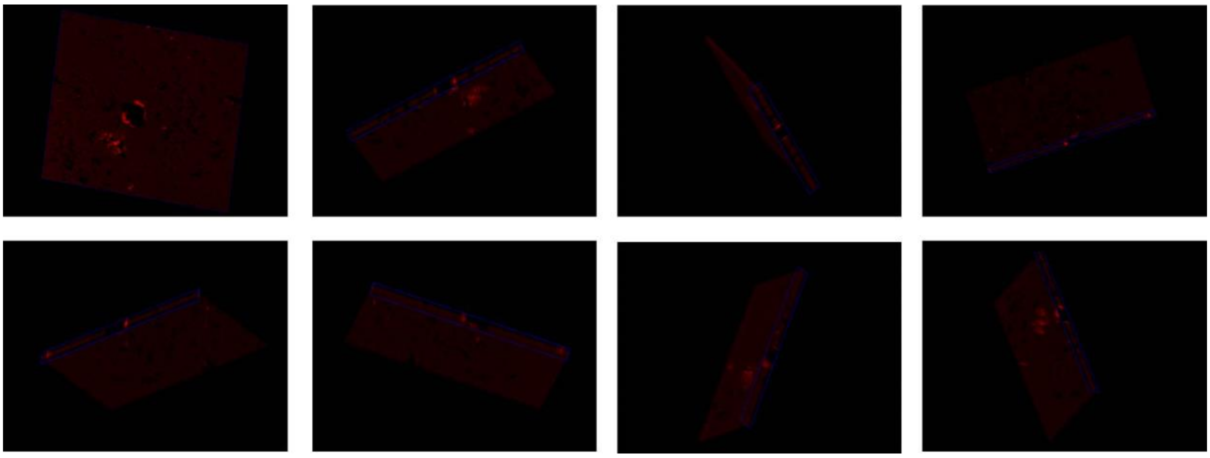

Supplementary Fig. 9. 3D scanning CFM images of a MFM-100 single crystal at different Z depths.

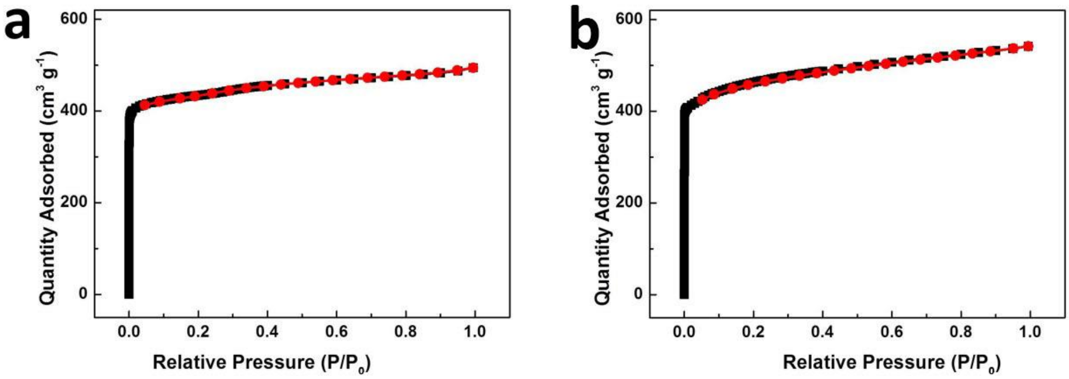

Supplementary Fig. 10. N_2 adsorption-desorption isotherms. (a) MFM-100a; (b) MFM-100b.

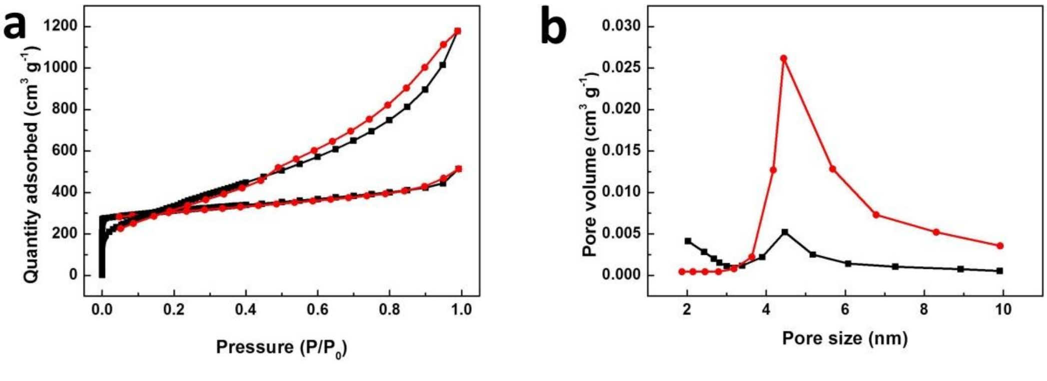

Supplementary Fig. 11. BET measurements of MFM-100c and MFM-100d. (a) N_2 adsorption-desorption isotherms; (b) mesopore size distribution.

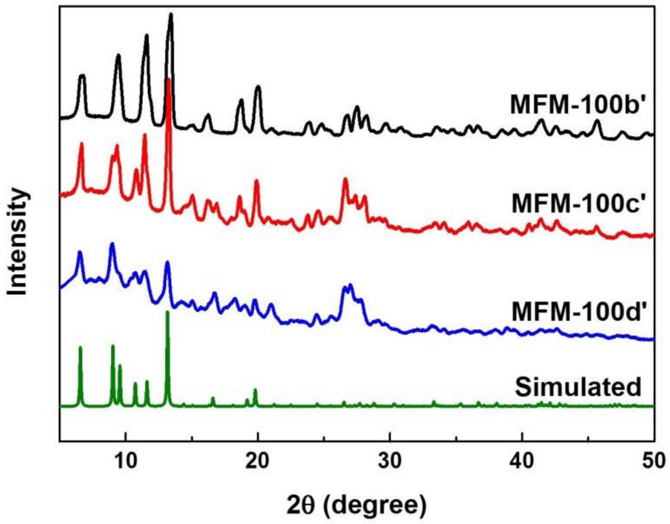

Supplementary Fig. 12. PXRD patterns of MFM-100(b’,c’,d’).

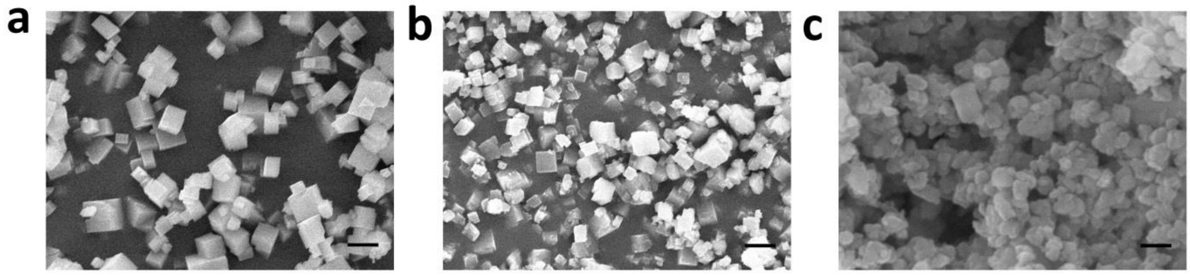

Supplementary Fig. 13. SEM images of (a) MFM-100b’, (b) MFM-100c’, and (c) MFM-100d’. The scale bars are 5 µm, 5µm and 300 nm respectively.

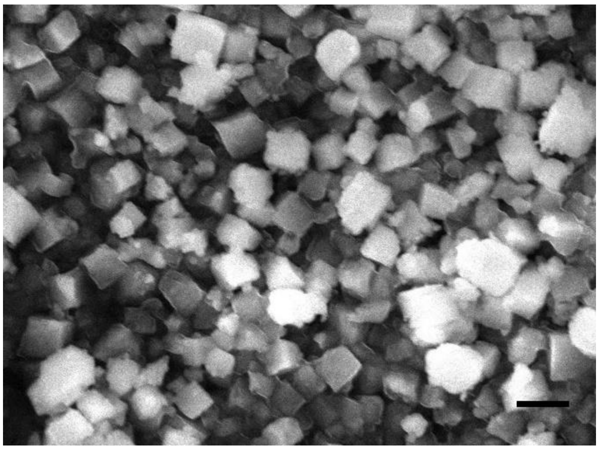

Supplementary Fig. 14. SEM image of MFM-100d’’. The scale bar is 3 µm.

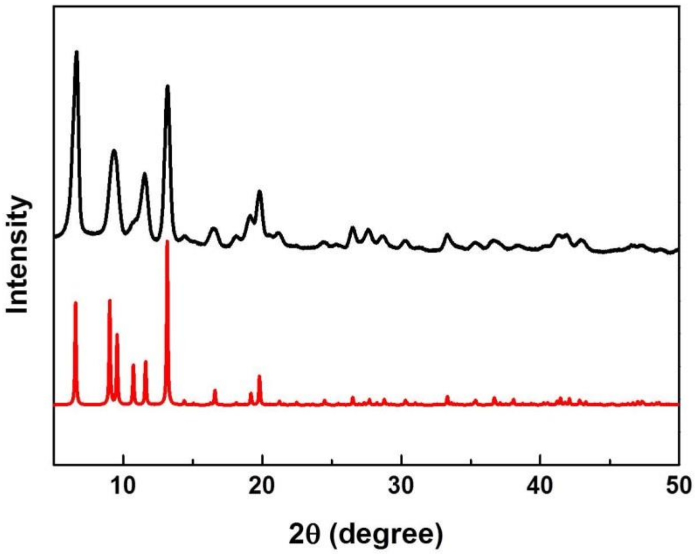

Supplementary Fig. 15. PXRD pattern of MFM-100d''. Black: experimental; red: simulated.

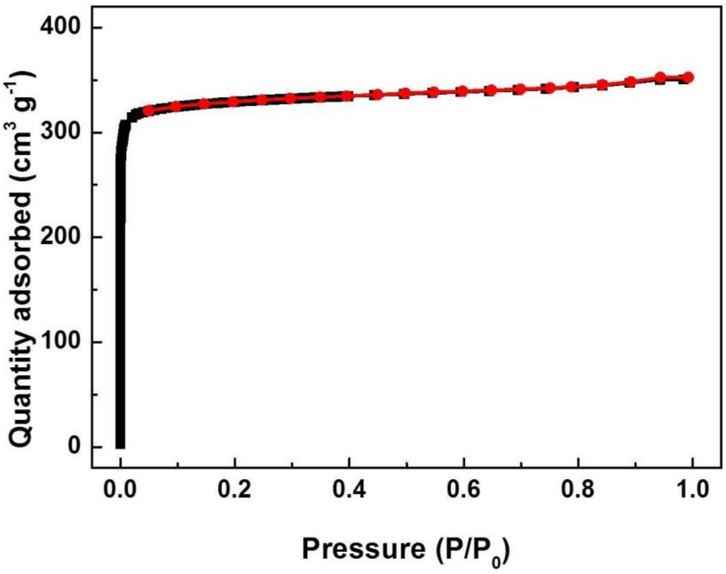

Supplementary Fig. 16. N_2 adsorption and desorption isotherm of MFM-100d''.

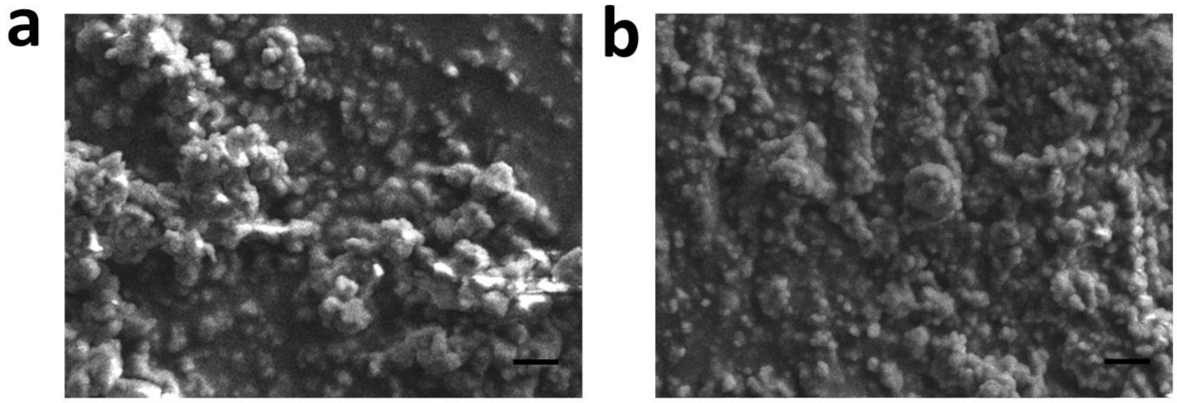

Supplementary Fig. 17. SEM images of (a) MFM-100d_{6V} and (b) MFM-100d_{10V}.

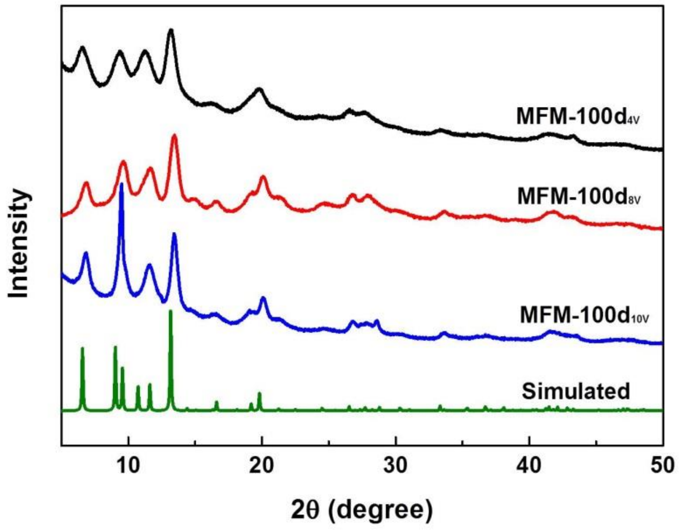

Supplementary Fig. 18. PXRD patterns of MFM-100(d_{6V},d_{8V},d_{10V}). MFM-100d_{8V} = MFM-100d

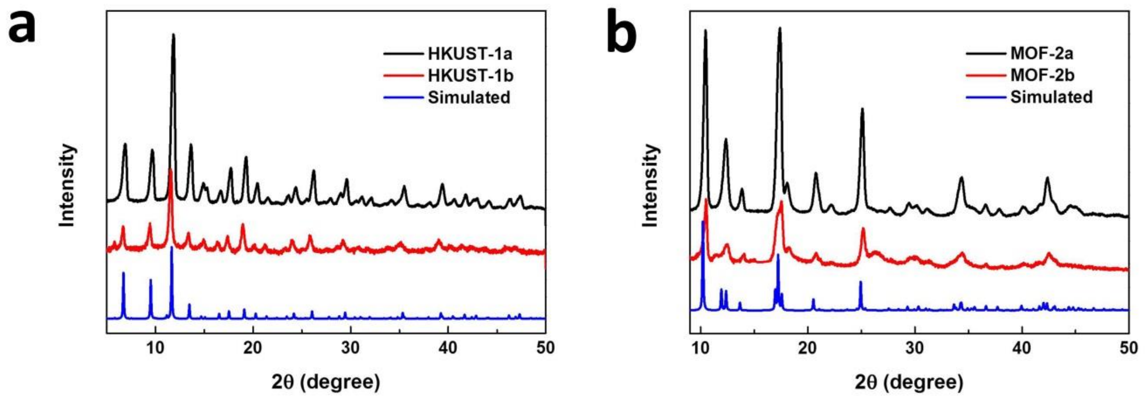

Supplementary Fig. 19. PXRD patterns. (a) HKUST-1; (b) MOF-2.

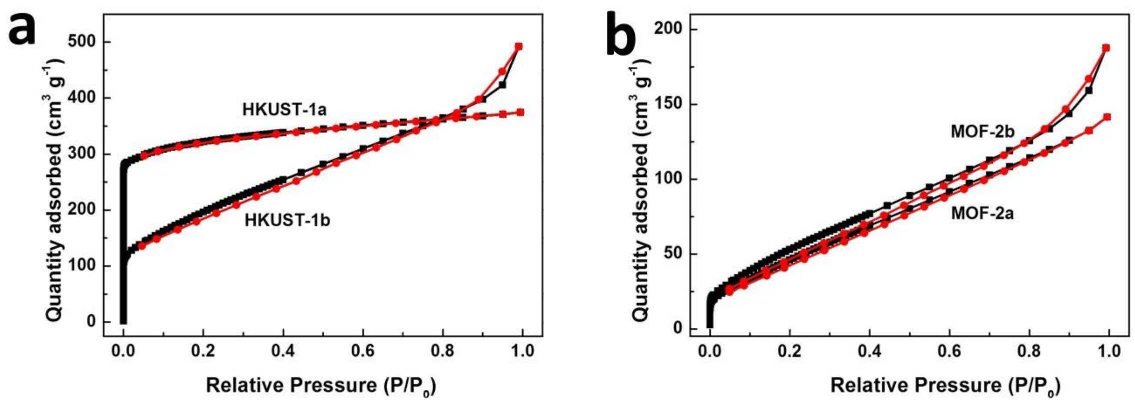

Supplementary Fig. 20. N_2 adsorption-desorption isotherms for (a) HKUST-1 and (b) MOF-2.

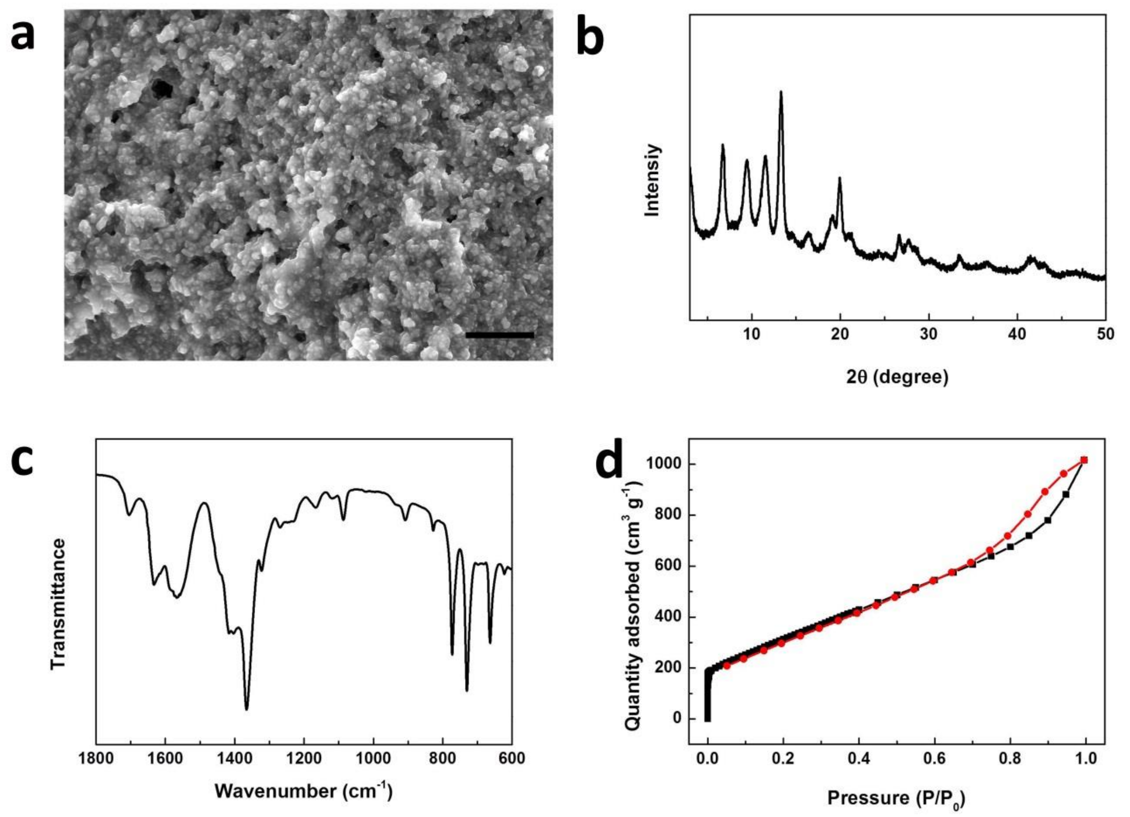

Supplementary Fig. 21. Characterization of used MFM-100d catalyst. (a) SEM image; (b) PXRD pattern; (c) IR spectrum; (d) N_2 adsorption/desorption isotherm at 77 K.

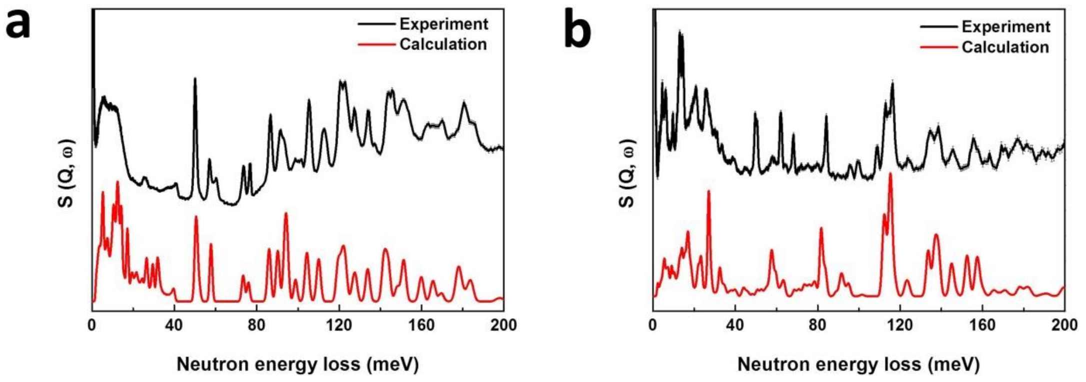

Supplementary Fig. 22. Comparison of calculated and experimental INS spectra of (a) benzyl alcohol and (b) MFM-100a.

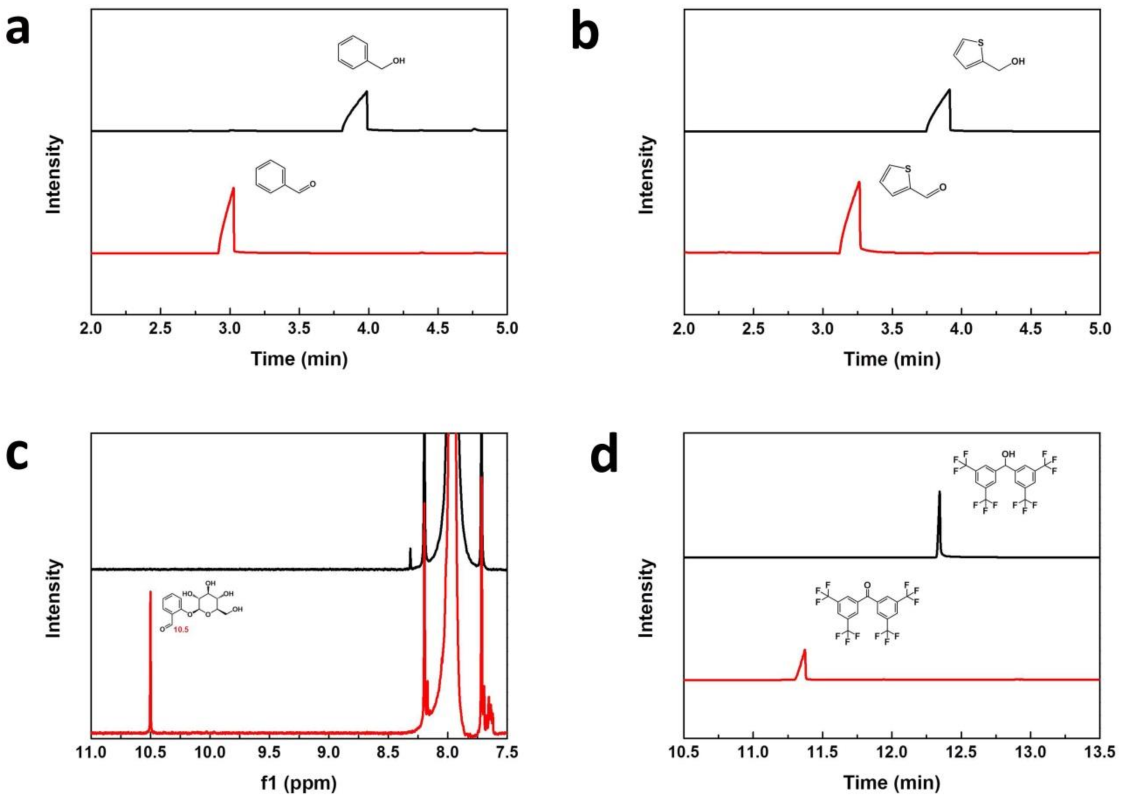

Supplementary Fig. 23. GC graphs of products from the oxidation of alcohols. (a) benzyl alcohol; (b) thiophen-2-ylmethanol; (c) salicin; (d) 3,3',5,5'-tetrakis(trifluoromethyl)benzhydrol.

Supplementary Tables

Supplementary Table 1. Elemental analytical data for samples of MFM-100.

Entry	Samples	N (wt%)	$Omim^+$ (wt%)*	Cu^{2+}/L^{4-} (mol/mol)†	$Omim^+/L^{4-}$ (mol/mol)‡	Cu^{2+}/L^{4-} (mol/mol) ⊥
1	MFM-100a	0	0	1.99	0	2.00
2	MFM-100b	0.24	1.67	1.97	0.06	1.97
3	MFM-100c	0.71	4.94	1.90	0.16	1.92
4	MFM-100d	1.58	11.00	1.79	0.48	1.76

*The $Omim^+$ content calculated based on ICP-OES results.
†The molar ratio of Cu^{2+}/L^{4-} calculated based on ICP-OES results.
‡The molar ratio of $Omim^+/L^{4-}$ calculated based on ICP-OES results.
⊥The molar ratio of Cu^{2+}/L^{4-} calculated based on ICP-OES results.

Supplementary Table 2. Comparisons of benzyl alcohol oxidation to benzaldehyde using different MFM-100 catalysts.

Entry	Catalysts	Yield (%)
1	MFM-100a	40
2	MFM-100b	51
3	MFM-100c	8
4	MFM-100d	>99
5	MFM-100a’	49
6	MFM-100b’	55
7	MFM-100c’	58
8	MFM-100d’	61
9	MFM-100d_{6V}	97
10	MFM-100d_{10V}	90

Reaction conditions: 0.1 mmol catalyst (based on Cu content from ICP); 1 mmol alcohol, 0.1 equiv TEMPO; 0.2 equiv K_2CO_3; 4 mL solvent (entries 1-12 MeCN, 13-24 DMF); 1 atm O_2; 75 ^oC; 3 h.

Supplementary Table 3. Comparisons of benzyl alcohol oxidation to benzaldehyde using different Cu-MOF catalysts.

Entry	Catalysts	T (°C)	Time (h)	Yield (%)	TOF (h^{-1})[‖]	Ref.
1[*]	MFM-100d	75	3	>99	3.33	This work
2[†]	HKUST-1	75	3	100	1.23	1
3[‡]	HKUST-1	75	22	89	0.16	2
4[⊥]	HKUST-1	70	9	94	2.09	3
5[⊣]	Cu-MOF-74	70	12	89	1.48	4

[*]0.1 mmol catalyst (based on Cu content from ICP); 1 mmol alcohol, 0.1 equiv TEMPO; 0.2 equiv K_2CO_3; 4 mL MeCN; 1 atm O_2.
[†]30 mg catalyst; 0.185 mmol alcohol; 0.5 equiv TEMPO; 1 equiv Na_2CO_3; 1 ml DMF; 1 atm O_2.
[‡]150 mg catalyst; 1 mmol alcohol; 0.5 equiv TEMPO; 1 equiv Na_2CO_3; 5 mL MeCN; 1 atm O_2.
[⊥]0.025 mmol catalyst; 0.5 mmol alcohol; 0.05 equiv TEMPO; 0.2 equiv NMI; 1 mL MeCN; 1 atm O_2.
[⊣]0.025 mmol catalyst; 0.5 mmol alcohol; 0.05 equiv TEMPO; 1 equiv DMAP; 1 mL MeCN; 1 atm O_2.
[‖]TOF is the turnover frequency, which was calculated as moles of converted benzyl alcohol per mole of Cu-MOF catalyst per hour.

Supplementary References

1. Peng, L. *et al.* Highly mesoporous metal-organic framework assembled in a switchable solvent. *Nat. Commun.* **5**, 4465 (2014).

2. Dhakshinamoorthy, A., Alvaro, M. & Garcia, H. Aerobic oxidation of benzylic alcohols catalyzed by metal-organic frameworks assisted by TEMPO. *ACS. Catal.* **1**, 48-53 (2011).

3. Kim, B. R., Oh, J. S., Kim, J. & Lee, C. Y. Robust aerobic alcohol oxidation catalyst derived from metal-organic frameworks. *Catal. Lett.* **146**, 734-743 (2016).

4. Kim, B. R., Oh, J. S., Kim, J. & Lee, C. Y. Aerobic oxidation of alcohols over copper-containing metal-organic frameworks. *Bull. Korean Chem. Soc.* **36**, 2799-2800 (2015).
